# Defocus Blur Detection and Estimation from Imaging Sensors

**DOI:** 10.3390/s18041135

**Published:** 2018-04-08

**Authors:** Jinyang Li, Zhijing Liu, Yong Yao

**Affiliations:** School of Computer Science and Technology, Xidian University, Xi’an 710071, Shannxi, China; liuzhijing@vip.163.com (Z.L.); yaoyong@xidian.edu.cn (Y.Y.)

**Keywords:** sparse representation, defocus blur, adaptive domain selection, compact dictionaries, nonlocal structure similarity, coefficients’ distributions

## Abstract

Sparse representation has been proven to be a very effective technique for various image restoration applications. In this paper, an improved sparse representation based method is proposed to detect and estimate defocus blur of imaging sensors. Considering the fact that the patterns usually vary remarkably across different images or different patches in a single image, it is unstable and time-consuming for sparse representation over an over-complete dictionary. We propose an adaptive domain selection scheme to prelearn a set of compact dictionaries and adaptively select the optimal dictionary to each image patch. Then, with nonlocal structure similarity, the proposed method learns nonzero-mean coefficients’ distributions that are much more closer to the real ones. More accurate sparse coefficients can be obtained and further improve the performance of results. Experimental results validate that the proposed method outperforms existing defocus blur estimation approaches, both qualitatively and quantitatively.

## 1. Introduction

Blur is an image degradation that commonly appears in consumer-level images obtained from a variety of image sensors [1,2,3,4]. Defocus blur is one type of blur degradation that results from defocus and improper depth of focus. For scenes with multiple depth layers, however, only the layer on a focal plane will focus on the camera sensor, which leads to others being out of focus. This phenomenon may sometimes strengthen a photo’s expressiveness, while, in most cases, it will lead to loss of texture details or incomprehensible information. In many scenarios, detecting and estimating the blur pixels can benefit a variety of image applications including but not restricted to image deblurring, image segmentation, depth estimation, objection recognition, scene classification and image quality assessment.

**Figure 1:**
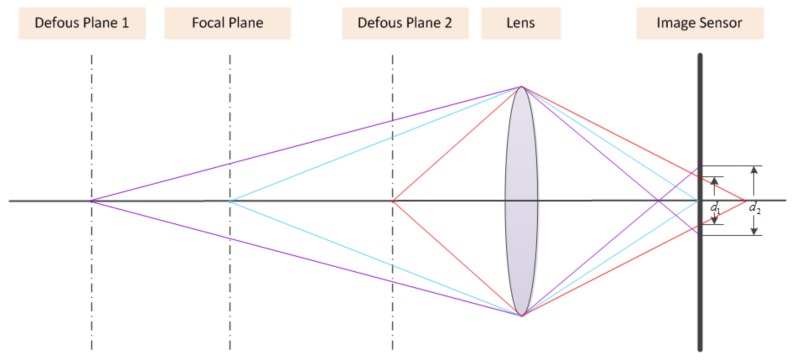
Focus and defocus for thin lens imaging sensors.

Assume that the defocus process can be modeled as a thin lens imaging system. Figure 1 illustrates the focus and defocus processes. Only the rays emitting from a focal plane will converge to a single point on a sensor plane and a sharp scene will appear, while the rays emitting from other planes will reach different points on a sensor plane and form circle regions. The circle region is called the circle of confusion (CoC) that results in defocus blur. From Figure 1, it is easy to verify that the larger the distance between the focal plane and the non-focal plane is, the greater the strength of defocus.

A number of methods for image blur analysis have been recently proposed; however, most of them focus on solving deblurring problems. On the contrary, there are a limited number of methods to explore defocus blur detection and estimation and the application is still far from practical. They assume that the defocus blur caused by multiple depth layers can be modeled by a latent image convolving with a spatial-variant kernel. In addition, the spatial-variant kernel is commonly assumed to be a disk or a Gaussian kernel. Therefore, the estimation of defocus blur map can be regarded as a deconvolution task. Cheong et al. [5] modeled a defocus blur kernel to be a Gaussian point-spread-function (PSF) and proved the amount of blur depends on the squared variance of the Gaussian PSF. In this method, the blur amount can be calculated from the first and second order derivatives. Oliveira et al. [6] defined the out-of-focus as a uniform disk kernel. This method is based on the assumption that the defocus blur kernel is characterized by its radius and can be provided by parametric models for each pixel efficiently. Zhang et al. [7] supposed that the defocus blur kernel to be a Gaussian function with standard σ and estimated the blur map by utilizing edges information. Then, a full blur map can be generated by utilizing a K nearest neighbors (KNN) matting method. The performances of these methods relay heavily on the accuracy of the PSFs, which is a challenging task in practical application.

There have been a series of methods proposed to handle a defocus blur problem. Conventional methods deal with defocus blur by utilizing a set of images of the same scene [8,9]. Using multiple focus settings, the defocus blur can be estimated during a deblurring process. Levin et al. [10] proposed modifying the aperture of the camera lens by inserting a patterned occluder so that they can recover a refocus image from a single input defocus image. Zhou et al. [11] used two coded apertures to complement each other and obtained a batter defocus blur measure for the two captured images. Xiao et al. [12] estimated a defocus blur kernel and restored a sharp image from time-of-flight depth camera. However, all of these methods require additional information or additional equipment that limit their applications in practice.

Since the level of defocus blur is intimately related with depth variation, depth information is of great value to defocus blur detection and estimation. A number of recently introduced methods aim at obtaining high quality in-focus images via scene depth from a single input image. Hu et al. [13] estimated and removed defocus blur from single images by utilizing depth estimation. However, the fine performance depended on the precisely separated depth layers and preassigned average depth of each layer, which may engender high computational cost and estimation error. Xiao et al. [12] proposed a joint optimization problem based on a model that the spatial-varying defocus blur kernel can be calculated for a given depth map. In this method, the defocus blur kernel matrix can be updated according to a currently estimated depth map.

In recent years, a variety of gradient and frequency based methods have been proposed to handle defocus blur analysis [14,15,16,17,18]. Elder and Zuker [19] firstly proposed a method for local blur estimation by utilizing the first and second order gradient information. Then, numerous methods have been proposed to detect and estimate defocus blur. Gradient-based methods [20,21,22] relied on a heavy-tailed distribution, which can be interpreted as an observation that the gradient distribution in a clear region should have more primarily small or zero gradient magnitudes. Frequency based methods [22,23] modeled defocus blur analysis exploiting the fact that the blur process decreases high-frequency components. Liu et al. [22] developed spectrum information and several blur features to classify blur images. Combining with the spectral and spatial domains, the method [24] utilized local power spectrum slope and total variation to assess image sharpness and estimate defocus blur. In [25], Shi et al. addressed the blur detection problem by constructing a combination of three local blur feature representations including image gradient distribution, Fourier domain descriptor, and local filters. Then, the blur map is formed in a discriminative way by utilizing a naive Bayesian classifier.

Sparse representation [26,27,28,29] has been known to be a very powerful statistical image modeling technique and successfully used in various low level restoration tasks. Shi et al. [30] has recently proposed a just noticeable defocus blur detection (JNB) method. However, it tends to inaccurately estimate the parametric distributions for the sparse coefficients and decrease the performance of defocus blur detection and estimation.

In this paper, a new method for blur detection and analysis is proposed. First, we use the principal component analysis (PCA) [31] technique to learn a set of compact dictionary and propose an adaptive domain selection scheme for sparse representation. Second, the proposed method learns nonzero-mean parametric distributions for coefficients based on the observation that nonzero-mean I.I.D Laplacian distributions do not fit the real coefficients’ distributions. Lastly, a blur strength measurement method is presented to evaluate the degree of defocus blur. Experimental results on various images show that the proposed method achieves better results than other approaches both in visual quality and evaluating indictor.

The paper is organized as follows. Section 2 introduces the general sparse representation models. Section 3 describes the details of adaptive domain selection, coefficient distributions learning and strength estimation for defocus blur detection and estimation, respectively. In Section 4, experimental results and comparison with other approaches are presented.

## 2. Blur Detection Model via Sparse Coding

Sparse representation is a powerful technique that has been widely used in signal processing or image restoration tasks. Recently, most of the approaches defined that natural images can be modeled with sparse representation over an over-complete dictionary. Using an over-complete dictionary $D\in R^{n\times l}$ that contains l dictionary atoms, an image patch can be represented as a sparse liner combination of these atoms

(1) y=Dα+n,

where $y\in R^{n}$ is a given image patch, D is an over-complete dictionary, α is a coefficient vector corresponding to D and n is a residual vector.

As illustrated above, $D\in R^{n\times l}$ is an over complete dictionary, which means that n<l. This problem becomes untraceable because many different coefficients give rise to the same y. Hence, additional information is required to constrain the solutions [32]. The sparsest representation coefficient has been proposed to be the solution

(2) $$\min_{\alpha }{∥\alpha ∥}_{0}\;s.t.\;y=D\alpha ,$$

or

(3) $$\min_{\alpha }{∥\alpha ∥}_{0}\;s.t.\;{∥y-D\alpha ∥}_{2}\leq \varepsilon ,$$

where ${∥•∥}_{0}$ is a $l_{0}$ norm that counts the number of the nonzero entries of vector α and ε is a small constant controlling the approximation error.

Equations (2) and (3) use a $l_{0}$ norm in the constraint and this induces sparsity and indicates that any signal can be described by a sparse number of dictionary atoms. However, a $l_{0}$ norm is nonconvex, which results in $l_{0}$-minimization of an NP-hard optimization problem. Thanks to [33], it has been proved that the $l_{1}$ norm is equivalent to the $l_{0}$ norm under certain conditions. Another sparse representation based method is proposed and can be expressed as

(4) $$\min_{\alpha }{∥\alpha ∥}_{1}\;s.t.\;{∥y-D\alpha ∥}_{2}\leq \varepsilon ,$$

where ${∥•∥}_{1}$ is a $l_{1}$ norm.

Besides the sparse coefficient, the selection of the dictionary also influences the performance of sparse representation based methods. The constructions of dictionary can be generally categorized into iteratively updating dictionary [34] and the universal one [32]. In the iteratively update construction manner, the minimizing model of Equation (4) involves simultaneously computing two variables: α and D. It can be solved by the alternating minimization scheme, which is commonly adopted when dealing with multiple optimization variables. In each iteration, dictionary D is fixed to estimate the coefficient α of each image patch

(5) $$\alpha^{\left(n+1\right)}=\text{arg min}{∥\alpha ∥}_{1}\;s.t.\;y={\bar{D}}^{\left(n\right)}\alpha ,$$

where $\alpha^{\left(n+1\right)}$ is the coefficient at iteration n+1 and ${\bar{D}}^{\left(n\right)}$ is the dictionary at iteration n.

Then, in the step of updating dictionary, each atom $d_{i}^{\left(n+1\right)}$ of dictionary D can be updated

(6) $$\begin{array}{cc} d_{i}^{\left(n+1\right)} & =\min∥y-D^{\left(n\right)}\alpha^{\left(n+1\right)}∥{}_{2}^{2}\\ & =\min_{d_{i}}∥y-\left(d_{i}\alpha {}_{i}^{\left(n+1\right)}+\sum_{j\neq i}d_{j}^{\left(n\right)}\alpha_{j}^{\left(n+1\right)}\right)∥{}_{2}^{2}\\ & =\min_{d_{i}}∥E_{i}^{\left(n+1\right)}-d_{i}\alpha {}_{i}^{\left(n+1\right)}∥{}_{2}^{2}, \end{array}$$

where $E_{i}^{\left(n+1\right)}=y-\sum_{j\neq i}d_{j}^{\left(n\right)}\alpha_{j}^{\left(n+1\right)}$ is the residual component.

In the universal construction manner, the K-SVD algorithm [32] designed a learning method based on the K-means clustering process and obtained over-complete dictionaries to achieve sparse signal representation. Given a set of training image patches, the K-SVD algorithm iteratively updates the sparse coding of the current dictionary and atoms of the dictionary.

## 3. Defocus Blur Detection and Estimation by Adaptive Domain Selection and Learning Parametric Distributions for Coefficients

In this section, the proposed method first presents the dictionaries learning method, which learns a series of compact dictionaries and adaptively assigns the optimal dictionary to each local patch as the sparse domain. All compact dictionaries are learned offline, and the proposed method online selects the dictionary. Then, we introduce sparse parametric distributions by nonlocal structural similarity for sparse coefficients. The improved method can be modeled as

(7) $$\min_{\alpha_{i}}{∥\frac{\alpha_{i}-\beta_{i}}{\theta_{i}}∥}_{1}\;s.t.\;{∥y_{i}-D_{k_{i}}\alpha_{i}∥}_{2}\leq \varepsilon ,$$

where $y_{i}$ is a patch extracted from an input defocus image and each patch is vectorized as a column vector of size n×1. $D_{k_{i}}$ is the optimal compact dictionary that is adaptively selected for the given patch $y_{i}$. The training method for $D_{k_{i}}$ is described in Section 3.1. $\alpha_{i}$ is the sparse coefficient for patch $y_{i}$ over $D_{k_{i}}$. $\beta_{i}$ and $\theta_{i}$ denote the mean and standard derivation for $\alpha_{i}$, respectively. In addition, ε is a small constant.

### 3.1. Sparse Representation by Adaptive Domain Selection

The sparse representation based approaches can achieve better performance in image restoration applications. However, many sparse decomposition models rely on learning an universal and over-complete dictionary to represent all image structures. The structures and contents vary remarkably across different images or different patches in a single image and the universal dictionary cannot satisfy all circumstances for defocus blur detection via sparse representation. In addition, it has been proved that sparse decomposition over a set of highly redundant basis is potentially unstable [35]. Therefore, an improved defocus blur estimation scheme, which prelearns a set of compact dictionaries, and adaptively assigning optimal dictionary to each local patch is proposed.

In order to learn the compact dictionary set for representing image structures, we first construct a dataset of blur local image patches by collecting images slightly blurred by Gaussian kernel with standard deviation σ=2.5 and cropped from them a rich amount of patches with size $\sqrt{n}\times \sqrt{n}$. Let $W=\left[w_{1},w_{2}\cdots w_{M}\right]\in R^{n\times M}$ be selected M blurred image patches. For better training performance, only pitches whose intensity variance, denoted by $\mathrm{IntVar}(w_{i})$, that are within the range of $\Theta_{1}$ and $\Theta_{2}$, i.e., $\Theta_{1}<\mathrm{IntVar}\left(w_{i}\right)<\Theta_{2}$, are selected.

In order to adaptively assign a dictionary to each local patch, the proposed method learns K compact dictionaries $D_{k}$ from the patch set W and generate K clusters from the patch set W by utilizing the K-means algorithm. Then, a dictionary can be learned from each of the K clusters and represent K distinctive patterns by the K dictionaries. Denote by $\left\{C_{1},C_{2},\cdots C_{K}\right\}$ the K clusters and $\mu_{k}$ the centroid of cluster $C_{k}$. Meanwhile, K subsets $W_{k}$ are obtained by partitioning W, where $W_{k}$ is a matrix of dimension of $n\times l_{k}$ and $l_{k}$ is the number of patches in $W_{k}$.

Now, we aim to learn a dictionary $D_{k}$ from the cluster $W_{k}$, which indicates that all the elements in $W_{k}$ can be exactly represented by $D_{k}$. Typically, the learning model can be formulated as

(8) $$\left({\tilde{D}}_{k},{\tilde{A}}_{k}\right)=\underset{D_{k},A_{k}}{\arg\min}\;∥W_{k}-D_{k}A_{k}∥{}_{F}^{2}+\lambda {∥A_{k}∥}_{1},$$

where ${∥•∥}_{F}$ is Frobenius norm and $A_{k}$ denotes the coefficient matrix of $W_{k}$ over dictionary $D_{k}$. λ denotes a parameter that balance the relationship between the data fidelity term and the regularization term. Minimizing model of Equation (8) involves simultaneously computing two variables: $A_{k}$ and $D_{k}$. It can be solved by the alternating minimization scheme, which is commonly adopted when dealing with multiple optimization variables.

However, utilizing Label (8) to learn the dictionary $D_{k}$ is stopped by some major issues. First, the optimizing task in Equation (8) involves simultaneously computing two variables: $D_{k}$ and $A_{k}$, which is computational challenging and time consuming. More importantly, the result of Equation (8) is commonly assumed to be an over-complete dictionary, which is redundant in the signal representing process and may not take advantage of similar patterns after K-means clustering. Specifically, $W_{k}$ is constructed via K-means clustering and can be treated as that all elements in $W_{k}$ share the similar patterns. Therefore, we prefer a compact dictionary rather than an over-complete one.

Here, the principal component analysis (PCA) [31] is applied to each subset $W_{k}$, so that each compact dictionary $D_{k}$ can be constructed via elements with similar pattern. Let $\Phi_{k}$ be the co-variance matrix of subset $W_{k}$. Then, the proposed method can obtain an orthogonal matrix $P_{k}$ by applying PCA to $\Phi_{k}$. For the purpose of reducing dimensionality of dictionary $D_{k}$, only the v eigenvectors corresponding to the first v largest eigenvalues in $P_{k}$ are selected to form the dictionary $D_{v}$. Denote by $D_{v}=\left[p_{1},p_{2},\cdots p_{v}\right]$ the constructed dictionary and let $A_{v}=D_{v}^{⊤}W_{k}$. It is obvious that a decrease of v will lead to an increase of data fidelity term $∥W_{k}-D_{v}A_{v}∥{}_{F}^{2}$ and a decrease of sparse term ${∥A_{v}∥}_{1}$. The optimal dimension $v_{r}$ of v can be determined as

(9) $$v_{r}=\underset{v}{\arg\min}\;∥W_{k}-D_{v}A_{v}∥{}_{F}^{2}+\lambda {∥A_{v}∥}_{1}.$$

In addition, $D_{k}=\left[p_{1},p_{2},\cdots p_{v_{r}}\right]$ is the compact dictionary corresponding to subset $W_{k}$.

**Figure 2:**
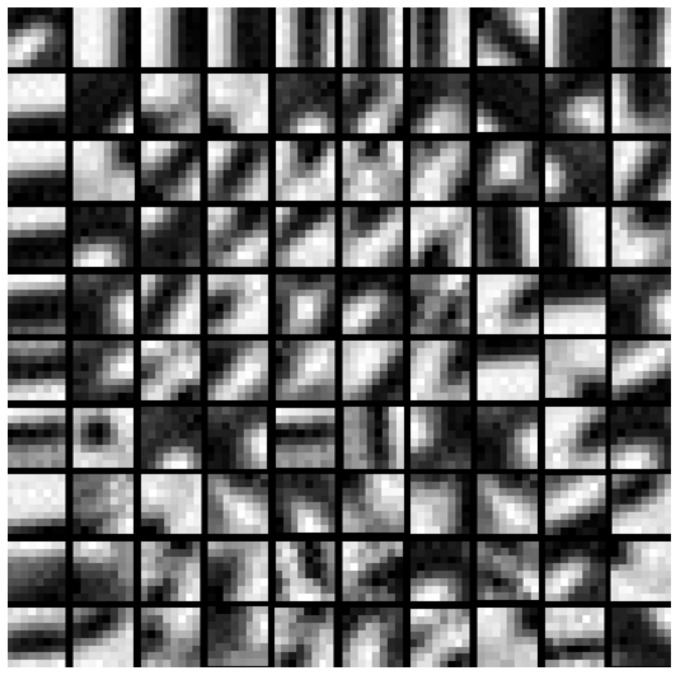
One example of the K compact dictionaries learned by PCA.

Following this procedure, all K compact dictionaries $D_{k}$ from K subsets $W_{k}$ can be obtained. Figure 2 shows an example of the learned dictionary from a training dataset.

With each compact dictionary $D_{k}=\left[p_{1},p_{2},\cdots p_{v_{r}}\right]$ learned, the proposed method can continue to assign an example $y_{i}$ to the most relevant compact dictionary in the dictionary set. Recall that the centroid $\mu_{k}$ is available, and the most relevant dictionary can be selected by

(10) $$k_{i}=\underset{k}{\arg\min}\;{∥y_{i}-\mu_{k}∥}_{2}.$$

### 3.2. Learning Coefficient Distributions with Nonlocal Structural Similarity

**Figure 3:**
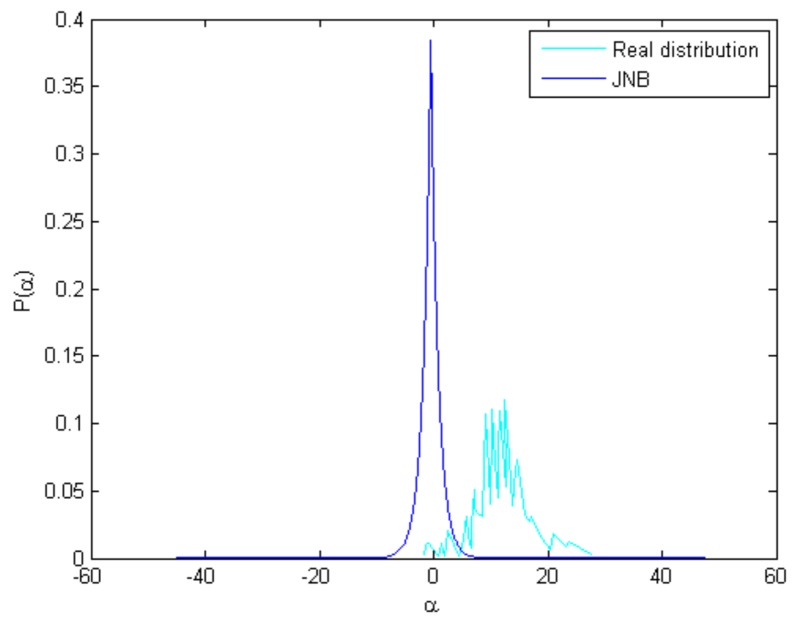
The observation of coefficients’ distributions between the JNB method and real distribution of a test image. The coefficients’ distributions are plotted by associating with the 4th atom of the dictionary that is learned by the K-SVD algorithm.

The JNB model [30] can achieve better results. However, due to the lack of nonlocal structural correlation [36], the JNB model tended to inaccurately estimate the parametric distributions for the sparse coefficients. It is easy to verify that the distribution of the common $l_{1}$ norm in Equation (4) equals an I.I.D zero-mean Laplaican distribution. Figure 3 shows the coefficients’ distributions obtained by the JNB method and real distribution of a test image. As illustrated in Figure 3, the I.I.D zero-mean Laplaican distribution can not fit the real coefficient distribution. Based on this observation, we generalise the nonlocal structural similarity and propose a nonzero-mean I.I.D Laplaican distribution to estimate the distribution of sparse coefficient for defocus blur detection.

First, the sparse model is extended based on the rich repetitive structures in blurred images. For each exemplar patch $y_{i}$, a patch set $Y_{i}=\left[y_{i,1},y_{i,2},\;\cdots ,y_{i,h}\right]\in R^{n\times h}$ is built via a patch matting algorithm in a larger window centered at i to group patches similar to $y_{i}$ (including $y_{i}$ itself). Each column of $Y_{i}$ corresponds to a patch similar to $y_{i}$. As the patches share similar structures, hence, we characterize the sparse representation of each patch in $Y_{i}$ as the same parametric distribution

(11) $$\min_{\alpha_{i,m}}{∥\frac{\alpha_{i,m}-\beta_{i}}{\theta_{i}}∥}_{1}\;s.t.\;{∥y_{i,m}-D_{k_{i}}\alpha_{i,m}∥}_{2}\leq \varepsilon ,$$

where m=1,2,⋯,h is the index from patch set $Y_{i}$. $y_{i,m}$ and $\alpha_{i,m}$ represent the $m^{\mathrm{th}}$ patch in patch set $Y_{i}$ and the corresponding sparse coefficient, respectively. $D_{k_{i}}$ denotes the pre-trained compact dictionary that adaptively selected for $y_{i,m}$, and $k_{i}$ can be obtained following Equation (10). ε is a small constant. $\beta_{i}$ and $\theta_{i}$ represent the mean and standard derivation, respectively.

Next, the nonlocal similar patches are used to accurately estimate the distribution parameters $\beta_{i}$ and $\theta_{i}$. The expectation of patch $y_{i}$ is estimated by

(12) $$\zeta_{i}=\sum_{m=1}^{h}w_{i,m}y_{i,m},$$

where $w_{i,m}=\left(1/c_{1}\right)\exp(-∥y_{i,m}-y_{i}∥/c_{2})$, wherein $c_{1}$ and $c_{2}$ denote the normalization coefficient and a predefined constant, respectively. Then, the more accurate mean $\beta_{i}$ is estimated as

(13) $$\beta_{i}=D_{k_{i}}^{⊤}\zeta_{i}=D_{k_{i}}^{⊤}\sum_{m=1}^{h}w_{i,m}y_{i,m}.$$

**Figure 4:**
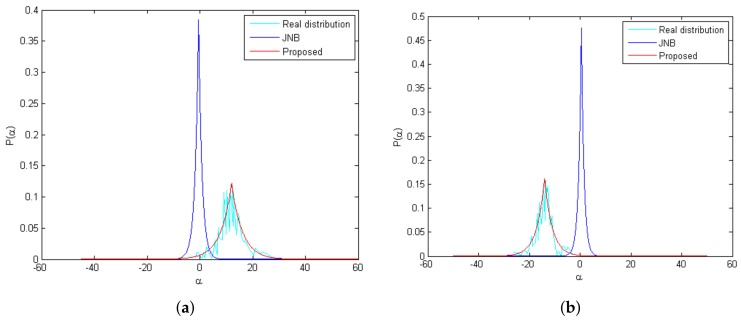
Comparison of the coefficients’ distributions of proposed method, the JNB method and the real distribution of the same test image; (**a**) the coefficients’ distributions associated with the 4th atom; (**b**) the coefficients’ distributions associated with the 6th atom.

With the grouped patch set and the mean $\beta_{i}$ estimated in Equation (13), the standard derivation $\theta_{i}$ for $\alpha_{i,m}$(m=1,2,⋯,h) can be estimated as

(14) $$\theta_{i}=\frac{1}{h}\sum_{m=1}^{h}{\left({\tilde{\alpha }}_{i,m}-\beta_{i}\right)}^{2}+\epsilon ,$$

where ${\tilde{\alpha }}_{i,m}=D_{k_{i}}^{⊤}y_{i,m}$, ϵ is a small positive number to ensure that $\theta_{i}$ is a non-zero value. Figure 4 shows the comparison of the coefficients’ distributions of the proposed method, the JNB method and the real distribution of the same test image. It is clear that the coefficients’ distributions learned by the proposed method is closer to the real distribution.

### 3.3. Strength Estimation for Defocus Blur

Denote by $s={∥\alpha_{i}∥}_{0}$ the sparse coefficient value. To estimate the strength of defocus blur, the proposed method first collects images with different blurriness levels. The images are blurred with the Gaussian kernel of standard deviation $\tilde{\sigma }$ ranging from 0.2 to 2.5. Then, the statistical relationships between the sparse coefficient value s and the corresponding blur standard deviation $\tilde{\sigma }$ can be obtained and fitted into a logistic regression function

(15) $$s=\frac{33.2071}{1+\exp(6.5125\tilde{\sigma }-4.1029)}+19.0774.$$

**Figure 5:**
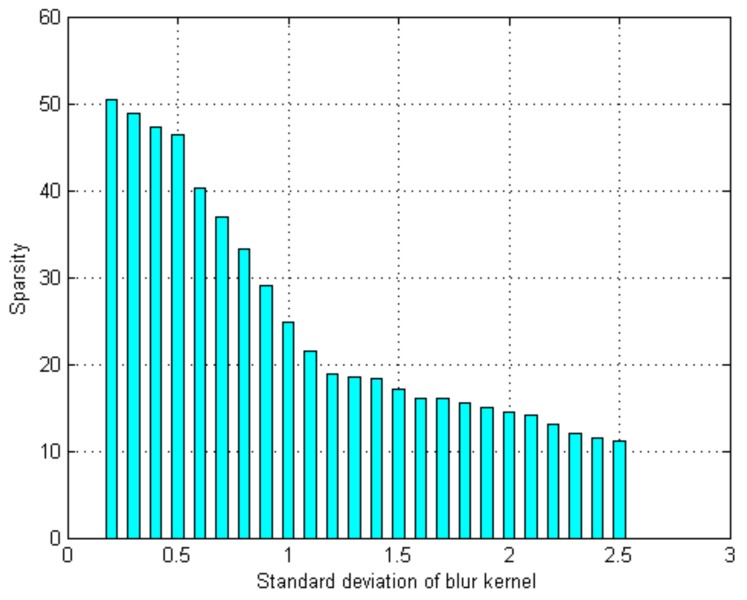
The association between sparsity of coefficient and blur strength.

Figure 5 shows the statistical relationships between the sparse coefficient value s and the corresponding blur standard deviation $\tilde{\sigma }$. With each sparse coefficient value calculated for an image patch, Equation (15) can be used to estimate the degree of defocus blur for each patch from a single defocus blur image.

## 4. Experimental Results

The performance of the proposed method was tested on defocus images dataset from [25]. The blurry regions in all tested images are masked out as ground-truth, which indicates the clear regions with respect to the defocus blur regions. In addition, the proposed method is also tested on 150 natural defocus blur images taken by consumer-level cameras or from the Internet with different defocus blur regions. Then, we compared the proposed method with several approaches including the JNB method [30], Vu’s method [24] and Shi’s method [25]. All the comparisons are performed by directly utilizing the public codes. In the experiments, each image patch is extracted with size 8×8 to form a 64D vector. The compact dictionary set is learned over 125,000 patches cropped from 1250 blurry images, which blurred by a Gaussian kernel with σ=2.5. The parameters of the proposed algorithm are set as follows: n=64, ε=0.175, M = 125,000, K=240 and h=24.

**Figure 6:**
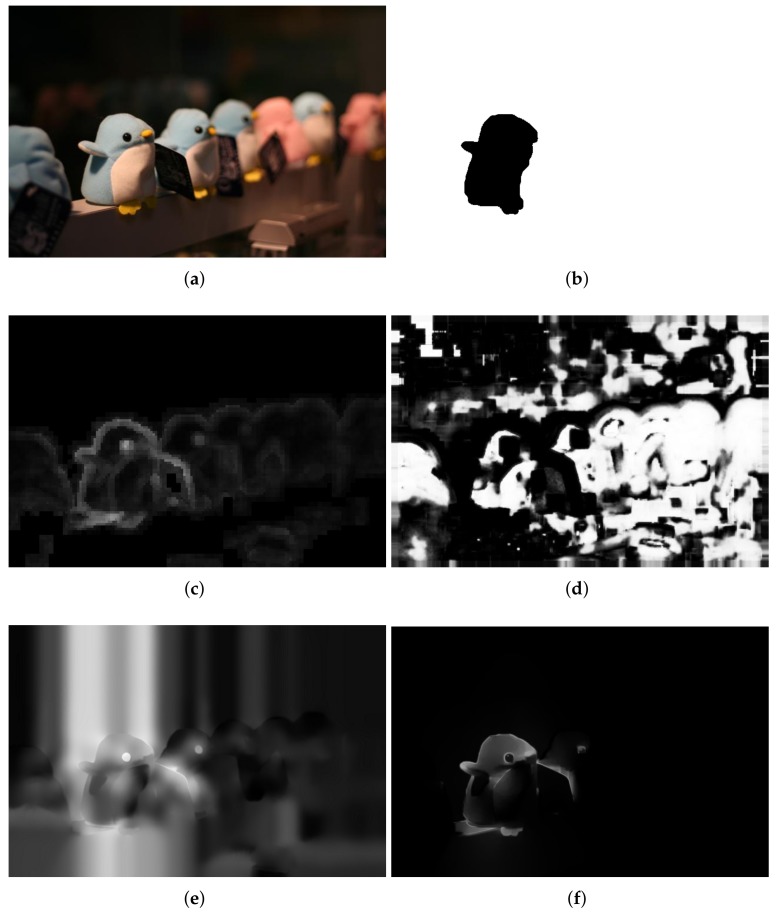
Comparison of different defocus blur detection and estimation using an input image form dataset [25], whose blur amount changes continuously. (**a**) the input blur image; (**b**) the ground-truth mask; (**c**) the detection and estimation results by Vu’s method; (**d**) the detection and estimation results by Shi’s method; (**e**) the detection and estimation results by the JNB method; (**f**) the detection and estimation results by the proposed method.

The performance of the proposed method was evaluated on the visual quality, the precision–recall (PR) and execution time. In the comparisons of visual quality, all of the compared results are normalized to [0, 1]. Figure 6 shows a set of experimental results using an input defocus blur image in which blur amount changes continuously. Vu’s method [24] combines both spectral and spatial sharpness to assess image sharpness. As shown in Figure 6c, Vu’s method [24] can roughly separate in-focus foreground from defocus background. However, it cannot handle flat regions and intends to smooth the boundaries because of total variation, such as facula and grass. Shi’s method [25] constructs a combination of three local blur feature representations including image gradient distribution, Fourier domain descriptor, and local filters. Then, the blur map is formed in a discriminative way by utilizing a naive Bayesian classifier. From Figure 6d, it shows that the results of Shi’s method [25] contain several estimation errors, which lead to a difficulty in separating the clear regions from the blur regions. In addition, a much longer processing time is required because of the combination of three local features, which cannot be satisfied in practical. Although the JNB method [30] can achieve a better result in detecting flat regions as shown in Figure 6e, it cannot detect defocus blur at strong edges’ regions and results in clear errors. The performance of the proposed method is shown in Figure 6f. The proposed method can result in much less artifacts and clear errors. Therefore, the proposed method performs better than the others both in separating clear regions from blur regions and representing details.

**Figure 7:**
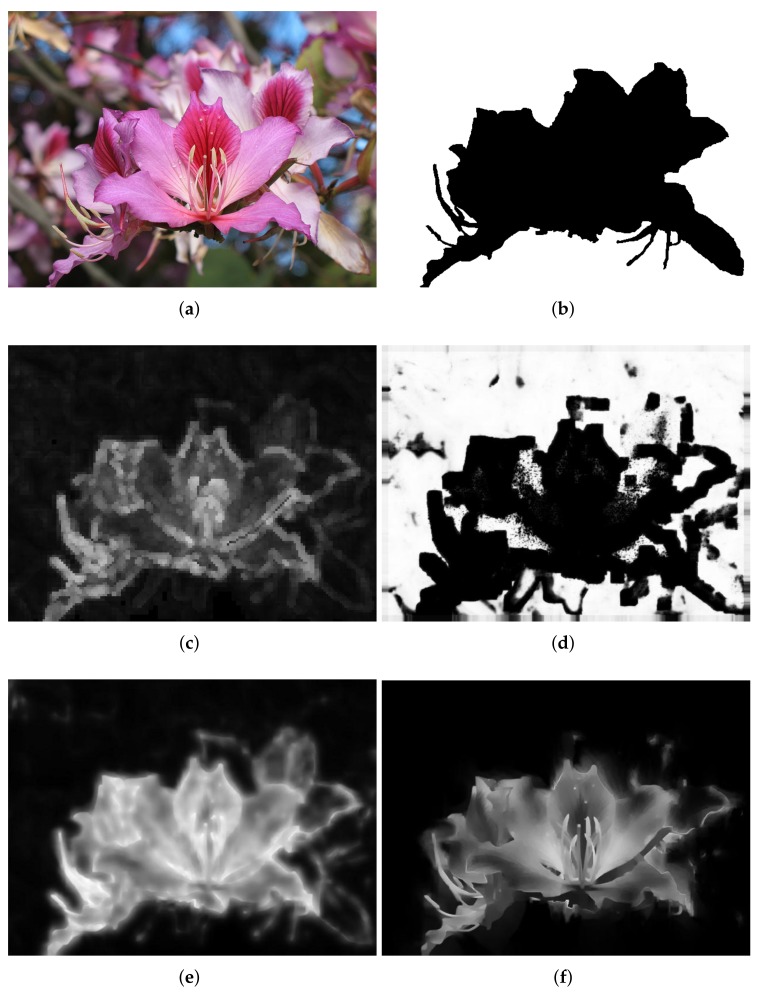
Comparison of different defocus blur detection and estimation using an input image form dataset [25], whose blur amount changes abruply. (**a**) the input blur image (Image source: originally posted to Flickr as Hong Kong Orchid tree flower); (**b**) the ground-truth mask; (**c**) the detection and estimation result by Vu’s method; (**d**) the detection and estimation result by Shi’s method; (**e**) the detection and estimation result by the JNB method; and (**f**) the detection and estimation results by the proposed method.

**Figure 8:**
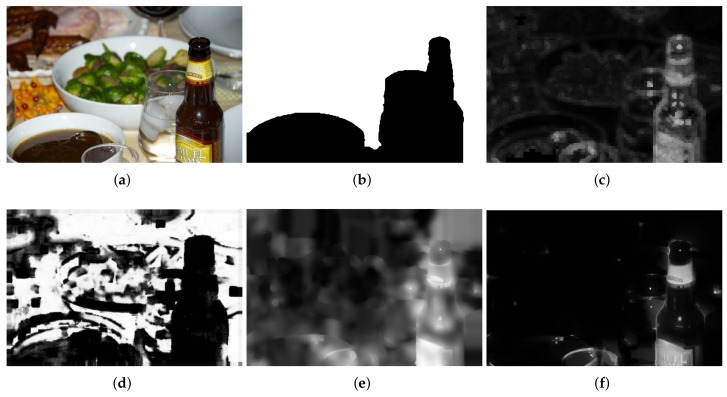
Comparison of different defocus blur detection and estimation using an input image form dataset [25], whose blur amount changes abruply. (**a**) the input blur image; (**b**) the ground-truth mask; (**c**) the detection and estimation result by Vu’s method; (**d**) the detection and estimation result by Shi’s method; (**e**) the detection and estimation result by the JNB method; (**f**) the detection and estimation results by the proposed method.

**Figure 9:**
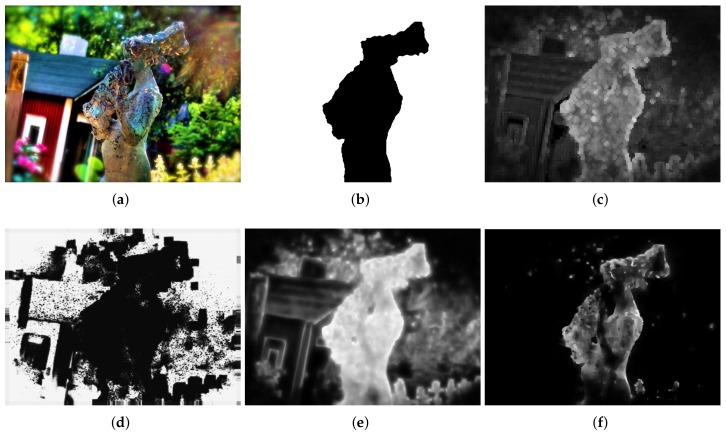
Comparison of different defocus blur detection and estimation using an input image form dataset [25], whose blur amount changes abruptly. (**a**) the input blur image; (**b**) the ground-truth mask; (**c**) the detection and estimation result by Vu’s method; (**d**) the detection and estimation result by Shi’s method; (**e**) the detection and estimation result by the JNB method; (**f**) the detection and estimation results by the proposed method.

Experimental results for defocus blur images whose blur amounts change abruptly are shown from Figure 7, Figure 8 and Figure 9. Vu’s method [24] assigns incorrect clear regions in the defocus blur regions of the background. The JNB method [30] contains too many clear errors and cannot produce significant differences between clear and blur regions. The proposed method is superior and the results of the proposed method are much closer to the ground-truth than that of others.

**Figure 10:**
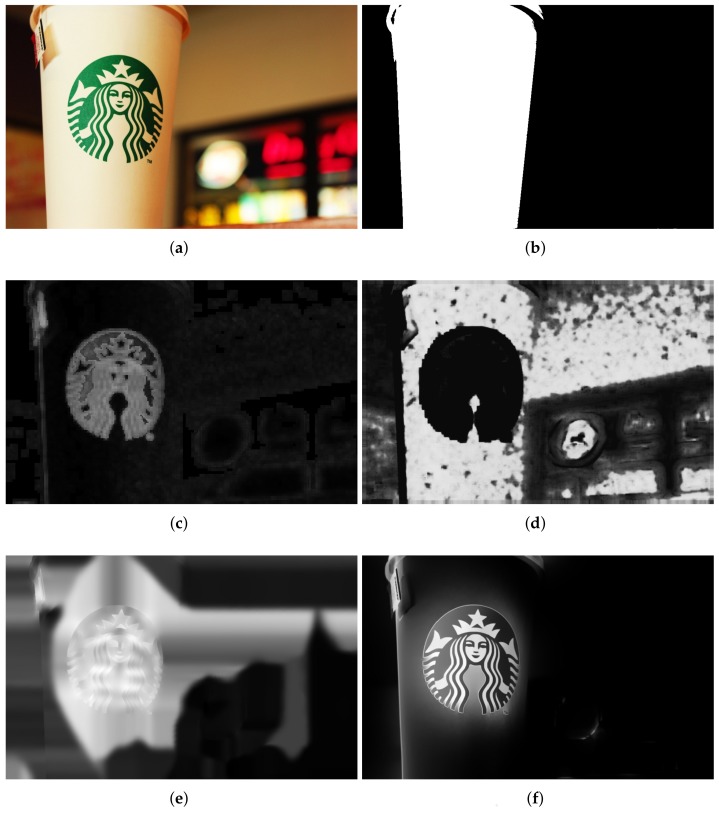
Comparison of different defocus blur detection and estimation using an input image form dataset [25], whose blur amount changes continuously. (**a**) the input blur image; (**b**) the ground-truth mask; (**c**) the detection and estimation result by Vu’s method; (**d**) the detection and estimation result by Shi’s method; (**e**) the detection and estimation result by the JNB method; (**f**) the detection and estimation results by the proposed method.

In additional, experimental results for defocus blurred image generated with a HUAWEI mobile phone are shown in Figure 10. Our method provides better detection and estimation performance than the others. Although successfully extracting the ground-truth from the blur regions, the method in Figure 10c also has errors in outliers’ regions. Figure 10d shows that the estimated local blur results have clear errors in separating the ground-truth from the blur regions. In Figure 10e, there are some artifacts in the result and the outline does not produce a significant difference to separate the ground-truth from the blur background. In contrast, Figure 10f shows that the proposed method produces favourable results in distinguishing ground-truth from the blur background regions and representing image details.

**Figure 11:**
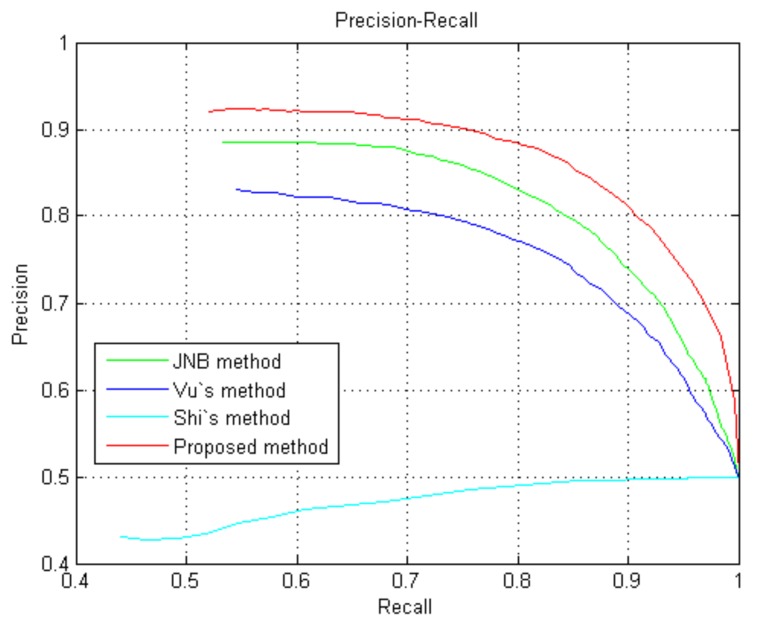
PR for different methods tested on defocus images from the dataset and naturally blurred images.

To further evaluate the effectiveness of the proposed method, we compare our method with other approaches via precision–recall (PR) in Figure 11. Forty defocus blur images (20 from dataset [25] and 20 from the naturally blurred images) are collected to test the proposed method. Figure 11 shows that the proposed method achieves the highest precision within almost the entire range of recall in [0, 1].

Table 1 shows the comparison of execution time by using images from the dateset [25]. All experiments were performed under the same computer configuration. The proposed method outperforms other defocus blur detection approaches by requiring much less computational time.

## 5. Conclusions

In this paper, we integrate the sparse representation model with adaptive domain selection and learning coefficient distribution for defocus blur detection and estimation. Compared with other defocus blur detection and estimation methods that rely on learning a universal and over-complete dictionary, the proposed method is helpful in adaptively selecting the optimal compact dictionary for each local patch and thus can much improve the accuracy and execution time of the defocus blur estimation. Based on the observation that the distributions of coefficients generally cannot be fitted with a I.I.D zero-mean Laplaican distribution, the proposed method learns parametric distributions from the gathered similar patches via nonlocal structural similarity. More accurate sparse coefficients can be obtained and further improve the the quality of the defocus blur detection. To estimate the strength of defocus blur, a criterion is defined to estimate the degree of defocus blur for each patch. Extensive experimental results show the superiority of the proposed method, both in visual quality and evaluation indexes.

## Figures and Tables

**Table 1:** Comparison of execution time (/s).

Data	Vu’s Method	Shi’s Method	JNB Method	Proposed Method
*out_of _ focus*0010	16.344	241.352	7.626	**5.473**
*out_of _ focus*0015	19.298	282.467	22.762	**16.251**
*out_of _ focus*0047	26.712	325.757	20.586	**19.329**
*out_of _ focus*0070	21.760	288.181	**7.794**	8.257
*out_of _ focus*0092	12.063	186.534	2.965	**2.019**
*out_of _ focus*0120	22.271	275.451	8.256	**5.060**
*out_of _ focus*0150	26.838	334.250	23.193	**14.225**
